# Hierarchical cross attention achieves pixel precise landslide segmentation in submeter optical imagery

**DOI:** 10.1038/s41598-025-08695-8

**Published:** 2025-07-01

**Authors:** Wenjie Hu, Guangtong Sun, Xiangqiang Zeng, Bo Tong, Zihao Wang, Xinyue Wu, Ping Song

**Affiliations:** 1 Institute of Disaster Prevention, Sanhe, 065201 China; 2 Hebei Key Laboratory of Resource and Environmental Disaster Mechanism and Risk Monitoring, Sanhe, 065201 China; 3 State Key Laboratory of Remote Sensing Science, Faculty of Geographical Science, Beijing Normal University, Beijing, 100091 China; 4 North China Institute of Science and Technology, Sanhe, 065201 China; 5 College of Physics and Optoelectronic Engineering, Ocean University of China, Qingdao, 266000 China

**Keywords:** Cross-attention, Landslide segmentation, Deep learning, Landslide, Geohazards, Environmental sciences, Natural hazards

## Abstract

Accurate landslide segmentation using remote sensing imagery is a critical component of geohazards response systems, particularly in time-sensitive tasks such as post-earthquake landslide damage assessment and emergency resource allocation. However, current methodologies struggle with two persistent challenges in sub-meter true-color imagery: fine-grained inter-class confusion between landslides and spectrally analogous terrain features, and within-landslide heterogeneity where localized damage signatures coexist with macro-scale deformation patterns within individual landslide bodies. To overcome these, we propose the Cross-Attention Landslide Detector (CALandDet), which improves the model’s ability to distinguish between landslide and background features by sharply capturing global landslide feature information and integrating global landslide feature information with local information via a cross-attention feature enhancement mechanism. Ablation experiments show that CALandDet outperforms baselines, as evidenced by a 4.89% enhanced *F*1 score and an 8.73% greater Intersection over Union (*IoU*). In comparative experiments, it outperforms the other models by 8.05–10.78% in *IoU* and 1.05–8.9% in *F*1 score, achieving an *IoU* of 82.65% and an *F*1 score of 81.64%. Furthermore, the Gradient-weighted Class Activation Mapping (Grad-CAM) visualizations confirm that the decision regions generated by the CALandDet model exhibit a higher spatial consistency with the actual landslide areas, effectively capturing indicative features including surface textures, sliding debris, accumulation bodies, and vegetation destruction. The proposed method may serve as a reference for future advancements in landslide segmentation and other remote sensing segmentation tasks.

## Introduction

As a prevalent natural hazard, landslides cause severe damage to communities, disrupting economic activities, destroying infrastructure, and endangering lives^1^. Landslide disasters are typically triggered by earthquakes or heavy rainfall, with large-scale landslides induced by strong earthquakes being particularly harmful^2^. According to relevant research, in some cases, the damage caused by landslides triggered by strong earthquakes can be more severe than the destruction caused by the earthquakes themselves^3^. Timely and precise identification of landslides within disaster chains is a critical step toward improving emergency response efficiency, facilitating real-time damage assessment, and informing science-based reconstruction planning^4^.

Traditional landslide detection methods primarily rely on field surveys and visual interpretation conducted by researchers. These approaches are not only highly susceptible to the subjective influences of expert knowledge but are also time-consuming and labor-intensive^1^. Thanks to the continuous advancements in computer science and hardware technology, several semi-automatic or automatic landslide detection methods based on remote sensing imagery have been proposed. For example, machine learning-based approaches such as artificial neural networks^5^support vector machines^6^and random forest^7^ are widely used to extract landslide features and generate landslide inventory maps. Despite advances in machine learning, existing methods for landslide identification remain constrained by labor-intensive feature engineering and workflow customization, posing significant challenges to systematic model improvement^8^.

**Fig. 1:**
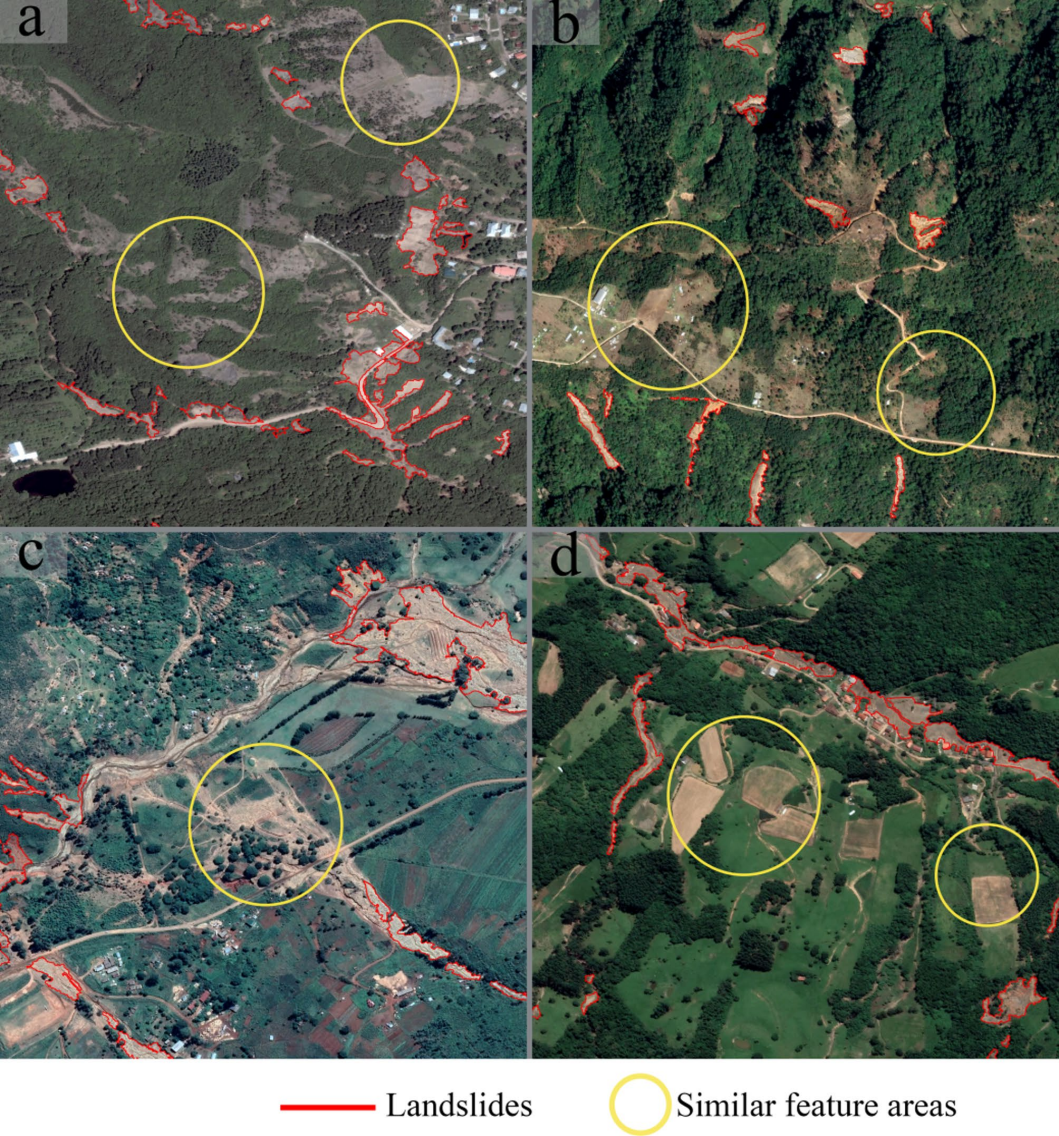
Similarity of features between landslides and backgrounds in true-color imagery. (**a**) Tbilisi, Georgia; (**b**) Tenejapa, Mexico; (**c**) Chimanimani, Zimbabwe; (**d**) Santa Catarina, Brazil. (Sourced from the publicly available landslide dataset released by Zhang et al.’s team^24^which the authors provide for researchers to use freely with only the requirement to cite the source (https://github.com/zxk688/GVLM). The visualization was implemented using Python 3.8.18 (https://www.python.org/), and the figure panels were laid out using Adobe Photoshop 22.5.4 (https://www.adobe.com/)).

The emergence of Convolutional Neural Network (CNN) has significantly advanced landslide detection by enabling automated feature extraction from high-resolution remote sensing imagery, overcoming limitations of manual methods reliant on expert-defined parameters^9^. A variety of CNN-based landslide segmentation models have been extensively proposed, including those based on Residual Networks^10^CNN models incorporating spatial-channel attention mechanisms^11^U-Net^12^PSPNet^13^and DeepLab V3 + ^14^. However, the locality and weight-sharing priors inherent in CNN, while effective for local pattern recognition, hinder their ability to model long-range dependencies between spatially dispersed landslide fragments (e.g., scarps and deposits), leading to incomplete semantic delineation in complex terrains^15^. To obtain a more comprehensive understanding of global features, researchers have applied Transformer architecture models to the field of landslide segmentation^16^. For example, SegFormer has shown superior performance in landslide segmentation compared to CNN^17^. Nevertheless, Transformer models are known for their computational complexity, which scales quadratically with the length of the input sequence, posing significant computational challenges^18^. To address this issue, the Swin Transformer^19^ has successfully reduced computational demands and enhanced image processing efficiency through a window-based attention mechanism. In the 2022 Landslide4Sense competition, a landslide segmentation model developed using the Swin Transformer architecture demonstrated outstanding performance and achieved remarkable results^20^. Additionally, models like CTDNet^21^MAST^22^and Swin-BiLSTM-FS^23^all based on the Swin Transformer architecture, have also performed well in the landslide detection field. As illustrated in Fig. 1, landslide areas and their surrounding backgrounds in true-color remote sensing imagery display remarkable visual similarities. This similarity in features greatly increases the complexity of landslide segmentation and presents significant challenges to accurate mapping. However, these methods have not been specifically designed to address the core challenge of sufficiently distinguishing landslide features from background features in complex remote sensing imagery.

Furthermore, the inherent opacity of deep learning architectures creates verification challenges in assessing whether algorithmic optimizations systematically align with geoscientific requirements^25^. In the application research of deep learning, the interpretability of models remains a critical issue that urgently needs to be addressed. Current deep learning models are generally characterized by their “black-box” nature, making it difficult for researchers to accurately understand the internal decision-making mechanisms and feature extraction processes, particularly in identifying the key decision regions that lead to the model’s final judgments^26^. This lack of interpretability directly impacts the evaluation of model performance, hindering researchers’ ability to determine whether the model is effectively learning and reasoning as intended^27^. By obtaining the decision regions of the model, researchers’ can better understand its working mechanisms and verify whether its recognition process aligns with the cognitive principles of the professional domain. This not only aids in assessing the reliability of the model but also provides a crucial foundation for its optimization and improvement. Therefore, interpretability is of paramount importance for landslide segmentation models.

Resolving these challenges necessitates that this study develop a landslide segmentation model based on a cross-attention feature enhancement mechanism. To validate the model’s accuracy and generalization performance, this research integrated the Bijie landslide dataset^11^the global very-high-resolution landslide mapping (GVLM) dataset^24^and the Sichuan high-precision aerial landslide mapping dataset (SCLM)^28^ to create a comprehensive landslide baseline dataset, testing the model’s ability to extract landslide information and its generalization performance. The main contributions of this study include:

1. We proposed a model named Cross-Attention Landslide Detector (CALandDet), specifically designed for landslide segmentation tasks. The model employs a Multilayer Perceptron Head (MLP Head) to progressively capture global landslide feature vectors and evaluate the landslide probability within samples. Subsequently, a cross-attention feature enhancement mechanism facilitates comprehensive interaction between the global landslide vectors and local feature maps. This mechanism enables the perception of landslide characteristics across diverse spatial scales while effectively suppressing interference from background noise. Evaluation results on the baseline dataset demonstrate that CALandDet outperforms other advanced landslide segmentation models in terms of reliability and generalization performance.
2. This study proposes a feature enhancement algorithm based on a cross-attention mechanism, designed to improve the model’s capability to discriminate between similar ground features, thereby enhancing the accuracy of landslide segmentation. The algorithm utilizes global feature vectors as its foundation and conducts feature information interaction calculations on feature maps through the construction of a cross-attention mechanism. Our ablation experiments show that adding the cross-attention mechanism greatly enhances the model’s recognition accuracy in landslide segmentation tasks, especially when it comes to telling the difference between landslides and other similar ground features.
3. This study employs the Gradient-weighted Class Activation Mapping (Grad-CAM) algorithm to visualize the decision regions of the model. By generating heatmaps of the decision regions, it provides researchers with an intuitive representation of the model’s decision-making process. Through comparative analysis of the heatmap distributions, the proposed algorithm in this study significantly enhances the model’s ability to focus on key feature regions of landslides. It can be observed that the improved model more accurately identifies typical landslide features while effectively suppressing interference from non-landslide areas.

The organization of this paper is as follows. Section 2 provides the processing of the research data; Sect. 3 describes the methods used in this paper and the details of the experiments; Sect. 4 presents the results of each method; Sect. 5 further analyzes the experiments; and finally, Sect. 6 presents the conclusions of this paper.

## Baseline dataset

### Data source

The Bijie landslide dataset^11^ has been developed and made publicly available by the research team at Wuhan University. This dataset is based on 0.8 m resolution optical imagery and encompasses 770 landslide incidents, including rock falls, rock slides, and a few debris slides, along with 2003 non-landslide area samples. Detailed information about the dataset and the download link can be accessed at: http://gpcv.whu.edu.cn/data/Bijie_pages.html.

The GVLM dataset^24^ was meticulously developed and released by the research team at Wuhan University of Science and Technology in 2023. This dataset, interpreted from Google Maps imagery at a resolution of 0.59 m, encompasses a broad range of landslide samples across 17 subregions globally. It includes various types of landslides differing in size, shape, occurrence time, spatial distribution, and land cover types, providing a high-resolution perspective for global landslide research. Detailed information about the dataset and the download link can be accessed at: https://github.com/zxk688/GVLM.

The SCLM dataset^28^ was developed and released by the team at the Sichuan Geomatics Center in 2022. This dataset, based on high-resolution aerial imagery ranging from 0.2 to 0.9 m, includes 59 landslide disaster samples and 48 debris flow disaster samples from the period of 2008 to 2020. These samples represent typical landslide and debris flow events in Sichuan Province and its adjacent areas, including major disaster zones from the 2008 Wenchuan earthquake, the 2013 Lushan earthquake, and the 2017 Jiuzhaigou earthquake, as well as regions along the Jinsha and Dadu Rivers. Detailed information about the dataset and the download link can be accessed at: https://www.scidb.cn/en/detail?dataSetId=803952485596135424.

### Baseline dataset construction process

**Fig. 2:**
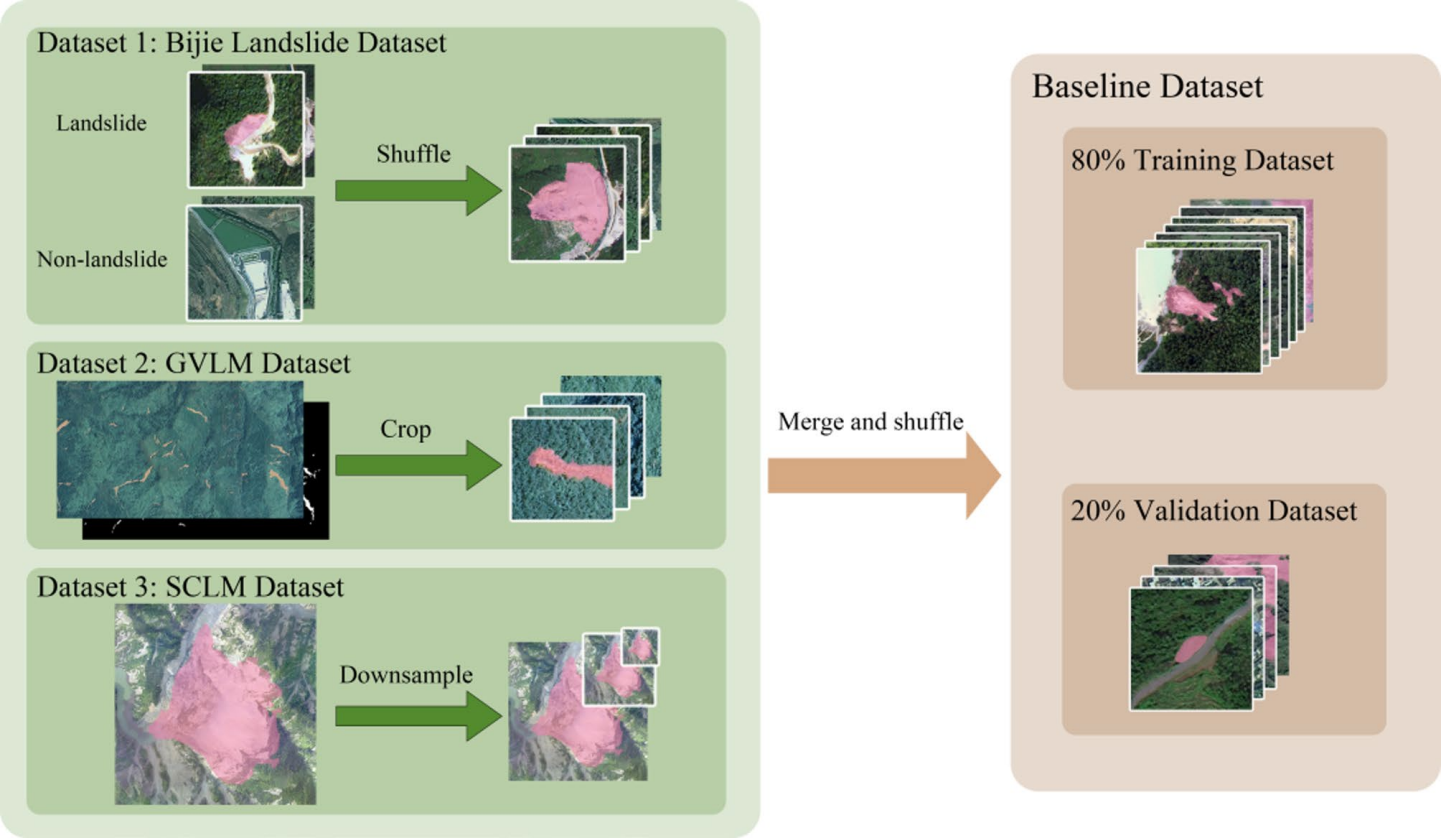
Baseline dataset construction process.

The methodological robustness of machine learning pipelines is fundamentally constrained by dataset representativeness^29^. Heterogeneous samples exhibiting multi-feature distributions not only enable rigorous stress testing of generalization boundaries but also establish essential baselines for comparative analysis of architectural efficacy under varying learning conditions^30^. To expand the dataset scale and enhance the diversity of sample features, thereby more comprehensively evaluating the model’s generalization learning capabilities, this study adopted a multi-source dataset fusion strategy.

As illustrated in Fig. 2, this study establishes a unified baseline dataset through integration of the Bijie landslide dataset^11^GVLM dataset^24^and SCLM dataset^28^. All images standardization to 224 × 224 resolution within a systematic preprocessing framework, ensuring dimensional compatibility with the model’s architectural specifications.

In this study, we considered differential preprocessing strategies were implemented based on dataset characteristics: analysis of the Bijie dataset revealed that 82.14% of landslide samples and 79.21% of non-landslide samples had dimensions below 300 and 400 pixels, respectively, demonstrating strong spatial compatibility with the target size. Consequently, an original-size preservation approach was adopted to prevent feature distortion, while random shuffling ensured balanced mixing of landslide and non-landslide samples, effectively eliminating spatiotemporal biases from data collection that could interfere with model training. For the GVLM large-scale imagery (1319 × 1946 to 7424 × 10808 pixels), a sliding-window cropping mechanism was employed to generate 300 × 300 pixel units, preserving spatial detail features. Regarding the SCLM high-resolution samples (1181 × 1181 pixels), 2× and 4× downsampling produced 580 × 580 and 256 × 256 pixel samples, respectively, establishing a cross-scale feature space to enhance the model’s scale-invariant recognition capability for landslide morphology.

Following the aforementioned processing steps, the three datasets were merged and randomly shuffled to construct a final baseline dataset comprising 8673 sample units. Statistical analysis revealed that landslide feature regions accounted for 7.0% of the total pixels, while non-landslide background regions constituted 93.0%. At the sample level, the dataset contained 3076 landslide samples and 5597 non-landslide samples. All the samples were randomly divided into a training set and a validation set in a 4:1 ratio. The training set included 6939 samples, with 2460 from landslide areas and 4479 from non-landslide areas. The validation set comprised 1734 samples, with 616 from landslide areas and 1118 from non-landslide areas. It is noteworthy that during the model training phase, oversized samples underwent random cropping to enhance data diversity by exploring potential feature combinations within redundant spatial regions, while undersized samples were subjected to position-adaptive random padding to improve the model’s robustness against positional variations of target features through spatial extension of the feature matrix. For the validation phase, oversized samples were processed via center cropping to match model input, whereas undersized samples were standardized using center padding to construct uniform input matrices. This method ensures that the model can make full use of the sample features during the training phase and that the evaluation is fair during the validation phase by using the same samples.

## Methodology

### Cross-attention feature enhancement mechanism

In order to address the effect of complicated geographical contexts on the accuracy of landslide detection, this work proposes a novel approach based on a cross-attention feature enhancement mechanism. The proposed framework aggregates multi-receptive-field features through cross-stage interactions between global geomorphological contexts and localized structural patterns. This dual-pathway architecture enables synergistic interaction, where the global contextual constraints guide the macroscopic identification of landslide-prone regions, while high-resolution local features capture fine-grained structural variations through trainable parameters. The global and local features fusion mechanism effectively suppresses spectral interference from non-landslide areas while enhancing discriminative feature representation along landslide boundaries and internal textures, thereby improving the model’s capability to distinguish geomorphologically similar terrain features and extract diagnostically critical landslide signatures.

**Fig. 3:**
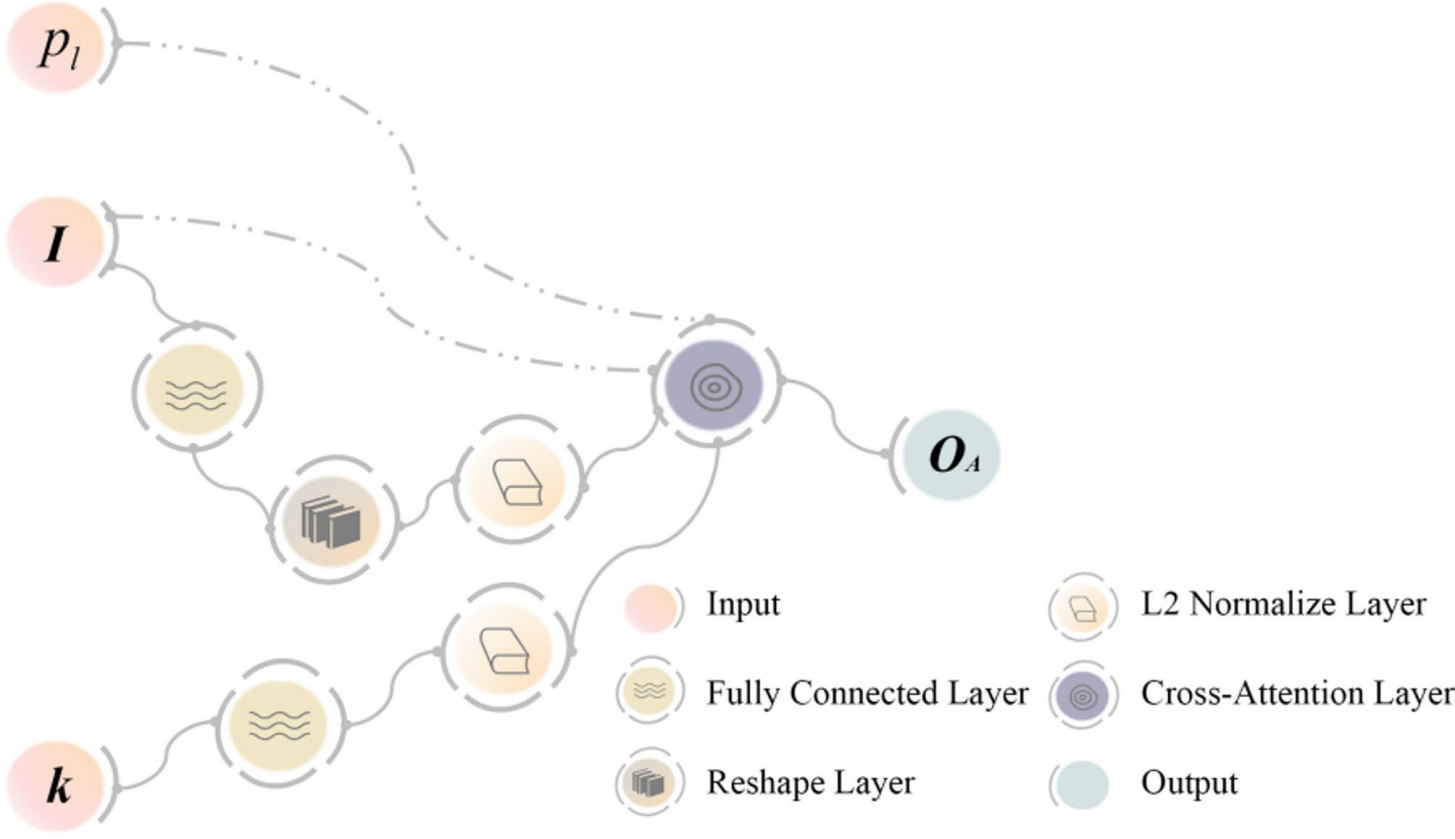
The schematic diagram of cross-attention feature enhancement mechanism.

Figure 3 shows the schematic diagram for this technique. This approach uses the feature map as the key and value for cross-attention computing and builds a global information vector with landslide features as the query. To accomplish spatial alignment of the feature information, the feature dimensions of the feature map ${\mathbf{I}}$ and the global feature vector ${\mathbf{k}}$ are first aligned, and both are then mapped to a hyper-sphere. To suppress background interference and amplify landslide-specific features, the correlation coefficients derived from dot product operations between the global landslide features map ${\mathbf{I}}$ and local feature vectors ${\mathbf{k}}$ are utilized for spatially adaptive feature recalibration. Lastly, the final output ${{\mathbf{O}}_{\mathbf{A}}}$ is obtained by constraining the cross-attention outcomes using the landslide relevance score ${p_l}$.

### Gradient-weighted class activation mapping

Gradient-weighted Class Activation Mapping (Grad-CAM)^31^ is a deep neural network visualization method based on gradient information. By calculating the gradient of the target class score with respect to the last feature map, it obtains the importance weights of the feature map and linearly combines them with the feature map to generate a class activation heatmap, thereby revealing the decision basis of the model^32^. Compared to traditional Class Activation Mapping (CAM)^33^ methods, Grad-CAM exhibits architecture independence, multi-modal compatibility, and fine-grained interpretability, making it suitable for various tasks such as image classification and visual question answering^34^. It also preserves the spatial resolution information of the feature map, providing precise visual explanations for model decisions. Mathematically, its essence can be described as a differential sensitivity analysis of the target output. Let the segmentation network function be $f({\mathbf{X}})={\mathbf{\hat {Y}}}$, where the input image ${\mathbf{X}} \in {{\text{R}}^{{\mathbf{H}} \times {\mathbf{W}} \times {\mathbf{C}}}}$ and the output segmentation map ${\mathbf{\hat {Y}}} \in {{\text{R}}^{{\mathbf{H}} \times {\mathbf{W}}}}$. For the landslide class probability ${\hat {y}_{p,q}}$ of any pixel $(p,q)$, its gradient sensitivity ${\alpha _k}$ on the last feature map ${\mathbf{A}} \in {{\text{R}}^{{\mathbf{h}} \times {\mathbf{w}} \times {\mathbf{k}}}}$ and the final decision region of the model are generated through channel-weighted summation ${{\mathbf{L}}_{{\mathbf{Grad-CAM}}}}$, defined as follows:

1 $${\alpha _k}=\frac{1}{Z}\sum\limits_{{i=1}}^{h} {\sum\limits_{{j=1}}^{w} {\frac{{\partial (\sum\limits_{{p=1}}^{H} {\sum\limits_{{q=1}}^{W} {{{\hat {y}}_{p,q}}} } )}}{{\partial {A_{i,j,k}}}}} }$$

2 $${{\mathbf{L}}_{{\mathbf{Grad-CAM}}}}=Relu(\sum\limits_{{k=1}}^{K} {{a_k}{{\mathbf{A}}^k}} )$$

where $Z=h \times w$ is the normalization factor, ${A_{i,j,k}}$ represents the activation value of the *k*-th channel at position $(i,j)$, and the $Relu( \cdot )$ function is used to retain positive contributions while suppressing negative responses. ${\hat {y}_{p,q}}$ represents the predicted value at the position $(p,q)$ output by the model. ${{\mathbf{A}}^k}$ denotes the activation feature map of the *k*-th channel, and *K* is the total number of feature maps.

### Model architecture

**Fig. 4:**
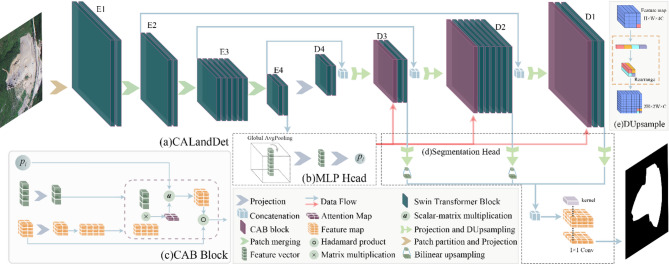
The proposed model architecture. (**a**) CALandDet; (**b**) MLP head; (**c**) cross-attention block; (**d**) segmentation head. (**e**) DUpsample (E*n* denotes the entire structure of Encoder stage *n*, D*n* denotes the entire structure of Decoder stage *n*).

The overall architecture of Cross-Attention Landslide Detector (CALandDet) is depicted in Fig. 4. In the design of the model, we introduced a Multilayer Perceptron Head (MLP Head) module to extract global features of the samples and compute their landslide relevance scores. These global features and scores are utilized in the subsequent Cross-Attention Block (CAB). As the core component of the model, the CAB integrates global features obtained from the MLP Head module with local information from the feature maps. By leveraging landslide relevance scores, it enhances the model’s robustness in identifying samples that contain only background, thereby improving its ability to finely distinguish between landslide features and background features. The CAB is methodically implemented at every network step after the DUpsampling^35^ operations, as seen in Fig. 4(a). DUpsample is a downsampling operation utilizing channel reorganization. It requires no additional learnable parameters and maximally preserves feature information, with its implementation shown in Fig. 4(e). A weighted fusion approach is used to gradually aggregate hierarchical characteristics through adaptive channel-wise recalibration after all processing stages are finished, producing refined landslide segmentation results, as shown in Fig. 4(d).

As shown in Table 1, the detailed parameter table of the model architecture specifies that Swin Transformer Block (STB) and Fully Connected Layer (FCL) represent their respective module types. Taking the stage 1 row as an example, the input to the E1 module is a 224 × 224 × 3 image, while the input to the D1 module comprises two sources—the 28 × 28 × 96 feature map output from the D2 module and the 28 × 28 × 192 feature map from E1 transmitted via skip connection. It should be noted that the input to the CAB module is not listed in the table, as its input is fixed as the 768-dimensional vector ${\mathbf{k}}$ and the scalar ${p_l}$. Regarding parameter notation, Conv2D: 4 × 4, 4, 96 defines a convolutional layer with a kernel size of 4 × 4, a stride of 4, and output channels of 96; FCL: [96, 48] indicates a fully connected layer with an input dimension of 96 and an output dimension of 48; CAB: [768, 48] denotes that its function is to map the input 768-dimensional vector ${\mathbf{k}}$ to a 48-dimensional space and involve it in cross-attention computation; and 2×STB: 7 × 7, 3 signifies that two STB modules are stacked at this stage, where each module has a window size of 7 × 7 and is configured with 3 attention heads.

**Table 1:** Architectural specifications.

Type	Input dims	Encoder	Decoder
Stage 1	I→224 × 224 × 3 D2→28 × 28 × 96 E1→28 × 28 × 192	Conv2D: 4 × 4, 4, 96 2×STB: 7 × 7, 3 FCL: [196, 192]	FCL: [96, 48] CAB: [768, 48] 2×STB: 7 × 7, 3
Stage 2	E1→28 × 28 × 192 D3→14 × 14 × 192 E2→14 × 14 × 384	2×STB: 7 × 7, 6 FCL: [192, 384]	FCL: [192, 96] CAB: [768, 96] 6×STB: 7 × 7, 6
Stage 3	E2→14 × 14 × 384 D4→7 × 7 × 384 E3→7 × 7 × 768	6×STB: 7 × 7, 12 FCL: [384, 768]	FCL: [384, 192] CAB: [768, 192] 2×STB: 7 × 7, 12
Stage 4	E3→7 × 7 × 768 E4→7 × 7 × 768	2×STB: 7 × 7, 24	FCL: [768, 384] 2×STB: 7 × 7, 24
MLP Head	E4→7 × 7 × 768	FCL: [768, 768] FCL: [768, 1]	
Segmentation Head	D4→56 × 56 × 48 D3→28 × 28 × 96 D2→14 × 14 × 192	FCL_D2_: [192, 16], FCL_D3_: [96, 16], FCL_D4_: [48, 16] Conv2D: 1 × 1, 1, 1	

#### MLP head

The spatial variability of landslide features and their semantic similarity to other surface features pose significant challenges for landslide segmentation. Resolving this problem requires, we designed an MLP Head block, whose primary task is to extract specific features ${\mathbf{k}}$ directly related to landslides from complex sample features, and to compute the landslide relevance scores ${p_l}$ for each sample. The direct coupling between this architectural block and the loss function enables efficient backward propagation of error gradients through the network backbone, thereby enhancing the feature extractor’s capacity to capture discriminative patterns within the data distribution during parameter optimization. This design strategy not only enhances the model’s capability to extract landslide features but also provides essential information for the subsequent CAB, thereby enabling the model to produce more precise and accurate landslide segmentation results. The architecture of the MLP Head is shown in Fig. 4(b).

Assuming the feature map ${\mathbf{I}} \in {{\text{R}}^{{\mathbf{H}} \times {\mathbf{W}} \times {\mathbf{C}}}}$ is input into the MLP Head module, we first compress it using a global average pooling layer to obtain the vector ${{\mathbf{k}}_{\mathbf{a}}} \in {{\text{R}}^{1 \times {\mathbf{C}}}}$, defined as follows:

3 $${{\mathbf{k}}_{\mathbf{a}}}={P_{avg}}({\mathbf{I}})$$

where ${P_{avg}}( \cdot )$ represents the operation performed by the global average pooling layer.

Subsequently, we input the vector ${{\mathbf{k}}_{\mathbf{a}}}$ into a fully connected layer consisting of *n* neurons for feature extraction and integration, thereby obtaining a global information-containing feature vector ${\mathbf{k}} \in {{\text{R}}^{1 \times n}}$, defined as follows:

4 $${\mathbf{k}}={F_{FC}}({{\mathbf{k}}_{\mathbf{a}}})$$

where ${F_{FC}}( \cdot )$ denotes a fully connected layer with *n* neurons, equipped with layer normalization and GELU activation.

Finally, we compute a value ${p_l}$, termed the landslide relevance score, by performing a weighted summation operation on the feature vector ${\mathbf{k}}$, as shown in Eq. (5). The model predicts the existence of a landslide when ${p_l}$ is positive, with higher values of ${p_l}$ indicating greater certainty. Conversely, if ${p_l}$ is negative, the model predicts the absence of a landslide; greater certainty in this prediction corresponds to more negative values of ${p_l}$.

5 $${p_l}={F_L}({\mathbf{k}})$$

where ${F_L}( \cdot )$ represents a linear layer containing a single neuron.

#### Cross-Attention block

Considering the ambiguity of landslide features in remote sensing imagery and the complexity of the background, this study has developed a CAB specifically designed for extracting landslide features. This block enhances landslide feature extraction and suppresses background noise interference. The CAB receives spectral feature maps along with global landslide features ${\mathbf{k}}$ and landslide relevance scores ${p_l}$ generated by the MLP Head. The CAB then assesses the correlation between various parts of the spectral feature maps and the global landslide features ${\mathbf{k}}$ to evaluate the likelihood of landslide presence in those parts. Subsequently, it adjusts the feature representations of different areas within the spectral feature maps, enhancing those with a higher probability of containing landslides while suppressing background areas. This process makes it possible for the model to efficiently combine local and global features: high-resolution local features use trainable parameters to capture fine-grained structural variations, while global features limit and directs the macro-scale identification of landslide areas, resulting in more accurate mapping results. The structure of the CAB is illustrated in Fig. 4(c).

The CAB module receives inputs from two branches: one is a spectral feature map ${{\mathbf{I}}_{{\mathbf{in}}}} \in {{\text{R}}^{H \times W \times C1}}$ with height *H*, width *W*, and feature dimension $C1$; the other is a feature vector ${\mathbf{k}} \in {{\text{R}}^{1 \times C2}}$ with dimension $C2$ and a landslide relevance score ${p_l}$, both outputted by the MLP Head block. In the initial stage of processing, we first align the spectral feature map ${{\mathbf{I}}_{{\mathbf{in}}}}$ and the feature vector ${\mathbf{k}}$ to the same feature dimension *d*. Thereafter, we merge the height *H* and width *W* dimensions of the processed feature map into a single dimension, resulting in ${{\mathbf{I}}_{\mathbf{d}}} \in {{\text{R}}^{n \times d}}$ and ${{\mathbf{k}}_{\mathbf{d}}}={{\text{R}}^{1 \times d}}$, here *n* equals the product of *H* and *W*. This is defined as follows:

6 $${{\mathbf{I}}_{\mathbf{d}}}={F_{merge}}({F_{FC}}({{\mathbf{I}}_{{\mathbf{in}}}}))$$

7 $${{\mathbf{k}}_{\mathbf{d}}}={F_{FC}}({\mathbf{k}})$$

where ${F_{FC}}( \cdot )$ represents a fully connected layer containing *d* neurons, equipped with layer normalization and GELU activation; ${F_{merge}}( \cdot )$ denotes the operation that merges the height *H* and width *W* dimensions of the feature map ${{\mathbf{I}}_{{\mathbf{in}}}}$ into a single dimension.

Considering the potential inconsistency in the feature spaces of ${{\mathbf{k}}_{\mathbf{d}}}$ and ${{\mathbf{I}}_{\mathbf{d}}}$, we employ *L*2 normalization to map each feature vector in ${{\mathbf{I}}_{\mathbf{d}}}$ and ${{\mathbf{k}}_{\mathbf{d}}}$ to the same feature space. The resulting vectors are ${{\mathbf{I}}_{{\mathbf{l2}}}} \in {{\text{R}}^{n \times d}}$ and ${{\mathbf{k}}_{{\mathbf{l2}}}} \in {{\text{R}}^{1 \times d}}$, defined as follows:

8 $${{\mathbf{I}}_{{\mathbf{l2}}}}=L2({{\mathbf{I}}_{\mathbf{d}}})$$

9 $${{\mathbf{k}}_{{\mathbf{l2}}}}=L2({{\mathbf{k}}_{\mathbf{d}}})$$

where $L2( \cdot )$ denotes the *L*2 normalization operation.

Next, we compute the dot product between ${{\mathbf{I}}_{{\mathbf{l2}}}}$ and ${{\mathbf{k}}_{{\mathbf{l2}}}}$ to obtain their attention feature map ${\mathbf{A}} \in {{\text{R}}^{n \times 1}}$. Subsequently, we decompose the *n*-dimensional attention feature map ${\mathbf{A}}$ back into the original dimensions *H* and *W*, resulting in ${{\mathbf{A}}_{\mathbf{R}}} \in {{\text{R}}^{H \times W \times 1}}$, which represents the global landslide cross-attention feature map of the original input ${{\mathbf{I}}_{{\mathbf{in}}}}$. Following this, we broadcast ${{\mathbf{A}}_{\mathbf{R}}}$ along the feature dimension to align it with the dimensions of ${{\mathbf{I}}_{{\mathbf{in}}}}$, resulting in ${{\mathbf{A}}_{\mathbf{B}}} \in {{\text{R}}^{H \times W \times C1}}$, defined as follows:

10 $${\bf{A}} = {{\bf{I}}_{{\bf{l2}}}} \cdot {{\bf{k}}_{{\bf{l2}}}}$$

11 $${{\bf{A}}_{\bf{R}}} = R({\bf{A}},n,H,W)$$

12 $${{\bf{A}}_{\bf{B}}} = B\left( {{{\bf{A}}_{\bf{R}}},C,{{\bf{I}}_{{\bf{in}}}}} \right)$$

where $R({\mathbf{A}},n,H,W)$ denotes the operation that splits the *n*-dimensional matrix ${\mathbf{A}}$ into the two dimensions *H* and *W*; $B\left( {{{\mathbf{A}}_{\mathbf{R}}},C,{{\mathbf{I}}_{{\mathbf{in}}}}} \right)$ denotes the broadcasting of ${{\mathbf{A}}_{\mathbf{R}}}$ along the *C* dimension to match the shape of ${{\mathbf{I}}_{{\mathbf{in}}}}$.

Considering the possibility that landslide regions may not be present in the samples, where the entire sample consists solely of background areas, the feature vector ${\mathbf{k}}$ generated by the MLP Head could reflect features irrelevant to landslides. This could potentially interfere with the normal functioning of the CAB. Therefore, we introduce a landslide relevance score ${p_l}$, which is used to constrain the output of the CAB module. Ultimately, the final result is obtained by performing the Hadamard product^36^ of ${{\mathbf{A}}_{\mathbf{R}}}$ and ${{\mathbf{I}}_{{\mathbf{in}}}}$, followed by multiplication with ${p_l}$, as calculated by Eq. (13).

13 $${{\mathbf{O}}_{{\mathbf{CAB}}}}{\text{=}}{p_l} \times {{\mathbf{I}}_{{\mathbf{in}}}} \circ {{\mathbf{A}}_{\mathbf{B}}}$$

where $\times$ denotes the scalar multiplication operator, and $\circ$ represents the Hadamard product operator.

### Experimental settings

#### Loss function

Considering the imbalance between landslide areas and background distribution in remote sensing images, we have use a loss function in this paper that combines cross-entropy loss^37^ and Dice loss^38^which can be expressed as Eq. (14). Cross-entropy loss evaluates pixel-wise classification discrepancies, while Dice loss measures overall landslide region differences. This combined local and global loss strategy enhances prediction accuracy under class imbalance.

14 $$\begin{aligned} L\left( {y,\hat {y},{p_l},{{\hat {p}}_l}} \right) & =1 - \frac{{2\sum\nolimits_{{i=1}}^{N} {{y_i}{{\left( {1+\exp \left( { - {{\hat {y}}_i}} \right)} \right)}^{ - 1}}} }}{{\sum\nolimits_{{i=1}}^{N} {{y_i}^{2}+\sum\nolimits_{{i=1}}^{N} {{{\left( {1+\exp \left( { - {{\hat {y}}_i}} \right)} \right)}^{ - 2}}+\varepsilon } } }} \\ & \quad - \sum\limits_{{i=1}}^{N} {\left( {{y_i}\log {{\left( {1+\exp \left( { - {{\hat {y}}_i}} \right)} \right)}^{ - 1}}+\left( {1 - {y_i}} \right)\log \left( {1 - {{\left( {1+\exp \left( { - {{\hat {y}}_i}} \right)} \right)}^{ - 1}}} \right)} \right)} \\ & \quad - \sum\limits_{{i=1}}^{N} {\left( {{p_l} \cdot \log {{\left( {1+\exp \left( { - {{\hat {p}}_l}} \right)} \right)}^{ - 1}}+\left( {1 - {p_l}} \right)\log \left( {1 - {{\left( {1+\exp \left( { - {{\hat {p}}_l}} \right)} \right)}^{ - 1}}} \right)} \right)} \\ \end{aligned}$$

where *y* represents the true labels, $\hat {y}$ is the model’s prediction output, ${y_i}$ is the value of the *i*-th pixel in the true labels, ${\hat {y}_i}$ is the value of the *i*-th pixel in the prediction output, *N* is the total number of pixels, $\varepsilon$ is a very small number used to avoid division by zero, ${p_l}$ is the probability of landslide presence according to the true labels, and ${\hat {p}_l}$ is the landslide relevance score output by the model.

#### Evaluation metrics selection

Considering the issue of imbalanced positive and negative samples in landslide identification tasks (where non-landslide areas account for > 90%), this study establishes an evaluation system for landslide identification centered on Intersection over Union (*IoU*) and *F*1 score, supplemented by Precision (*P*) and Recall (*R*) for multi-dimensional validation. *IoU* quantifies the spatial matching degree to effectively couple the identification accuracy of both positive and negative samples, while *F*1 score harmonizes the decision-making conflict between *P* and *R*. Together, they overcome the evaluation bias caused by sample imbalance. It must be emphasized that solely pursuing *P* values will lead to conservative predictions—for instance, identifying only high-confidence landslides may drive *P* toward 100%, resulting in missed detection of potential hazards. Conversely, focusing exclusively on *R* values will cause overfitting and generalization failure, such as when the entire image is classified as a landslide, yielding an *R* value of 100%.

Once the model’s basic performance is validated through *IoU* and *F*1 score, *P* and *R* can be strategically adjusted based on application scenarios: earthquake emergency response requires high *R* values to ensure comprehensive hazard coverage, supporting rescue route planning and damage assessment, whereas landslide susceptibility mapping demands high *P* values to guarantee sample reliability, providing an accurate training foundation for risk models.

The evaluation results of this study employ four metrics: *P*, *R*, *F*1 score, and *IoU*. These metrics are calculated based on the following four types of statistical data: true positives (*TP*), false positives (*FP*), false negatives (*FN*), and true negatives (*TN*). The corresponding formulas are presented as Eqs. (15) to (18).

15 $$P = \frac{{TP}}{{TP + FP}}$$

16 $$R = \frac{{TP}}{{TP + FN}}$$

17 $$F1 = \frac{{2 \cdot P \cdot R}}{{P + R}}$$

18 $$IoU = \frac{{TP}}{{TP + FP + FN}}$$

#### Implementation details

The runtime environment for this study is shown in Table 2, and the hyperparameter settings for the experiments are presented in Table 3.

**Table 2:** Runtime environment.

Configurations	
Hardware	
CPU	Intel(R) Core(TM) i9-10980HK CPU @ 2.40 GHz
GPU	NVIDIA GeForce RTX 3080 Laptop GPU
RAM	64.0 GB
Software	
OS	WSL 2 Ubuntu 22.04.2 LTS
Interpreter	PyCharm 2023.2.2 (Professional Edition)
Language	Python 3.11.5
CUDA toolkit	CUDA 12.4
Deep learning framework	PyTorch 2.1.0

**Table 3:** The hyperparameter setting of the study.

Hyperparameter	
Batch size	32
Sample size	224
Initial learning rate	1.0e−3
Epochs	200
Optimizer	AdamW^39^
Weight decay	0.05
Loss strategy	Online hard example mining^40^
Hard example mining rate	0.7
Training strategy	Warm-up^41^
Warm-up epoch	20
Learning rate scheduler	Cosine annealing learning rate scheduler^42^

## Results

### Ablation study

#### Cross-attention block and MLP head

**Fig. 5:**
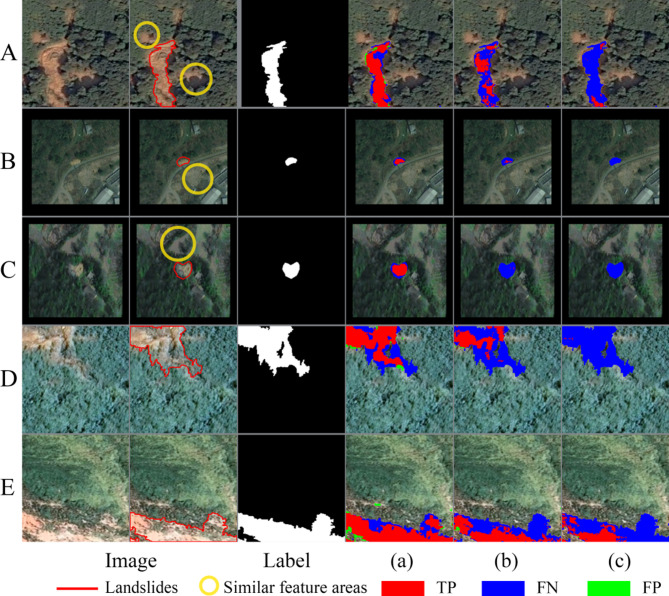
Visualization of ablation study results on the validation set samples. (**a**) Baseline + MLP Head + CAB; (**b**) Baseline + MLP Head; (**c**) Baseline (the visualization was implemented using Python 3.8.18 (https://www.python.org/), and the figure panels were laid out using Adobe Photoshop 22.5.4 (https://www.adobe.com/)).

In the ablation study section of this research, we trained the model using the training set of a baseline dataset and evaluated and compared the performance of various models using the validation set. Initially, we removed the MLP Head module and the CAB module from CALandDet to serve as the baseline model. Subsequently, different modules were incrementally added to this baseline model to assess their impact on performance, with specific results detailed in Table 4. Additionally, we visualized the results of the ablation study on the validation set, with relevant illustrations presented in Fig. 5. In subsequent analyses, we will pay particular attention to two metrics: the *IoU* and the *F*1 score, as they comprehensively reflect the overall performance of the model.

As shown in Table 4, compared to the baseline model, the addition of the MLP Head module in the Baseline + MLP Head model resulted in an increase of 0.52% in *IoU* and 2.21% in *F*1 score. The Baseline + MLP Head + CAB model, compared to the baseline model, achieved the highest improvements with an increase of 8.73% in *IoU* and 4.89% in *F*1 score.

**Table 4:** The evaluation results of the ablation study on the validation set.

Model name	*IoU* (%)	*F*1 (%)	*P* (%)	*R* (%)
Baseline	73.92	76.75	**87.44**	68.39
Baseline + MLP head	74.44	78.96	84.08	74.42
Baseline + MLP head + CAB	**82.65**	**81.64**	82.95	**80.38**

#### Visual comparison

As shown in Fig. 5, the Baseline + MLP Head architecture achieved partial improvements over the Baseline model in detecting narrow landslide features and indistinct morphological signatures: Samples A and D exhibited higher recognition accuracy, while sample B, despite incomplete segmentation of small targets, still achieved partial localization.

**Fig. 6:**
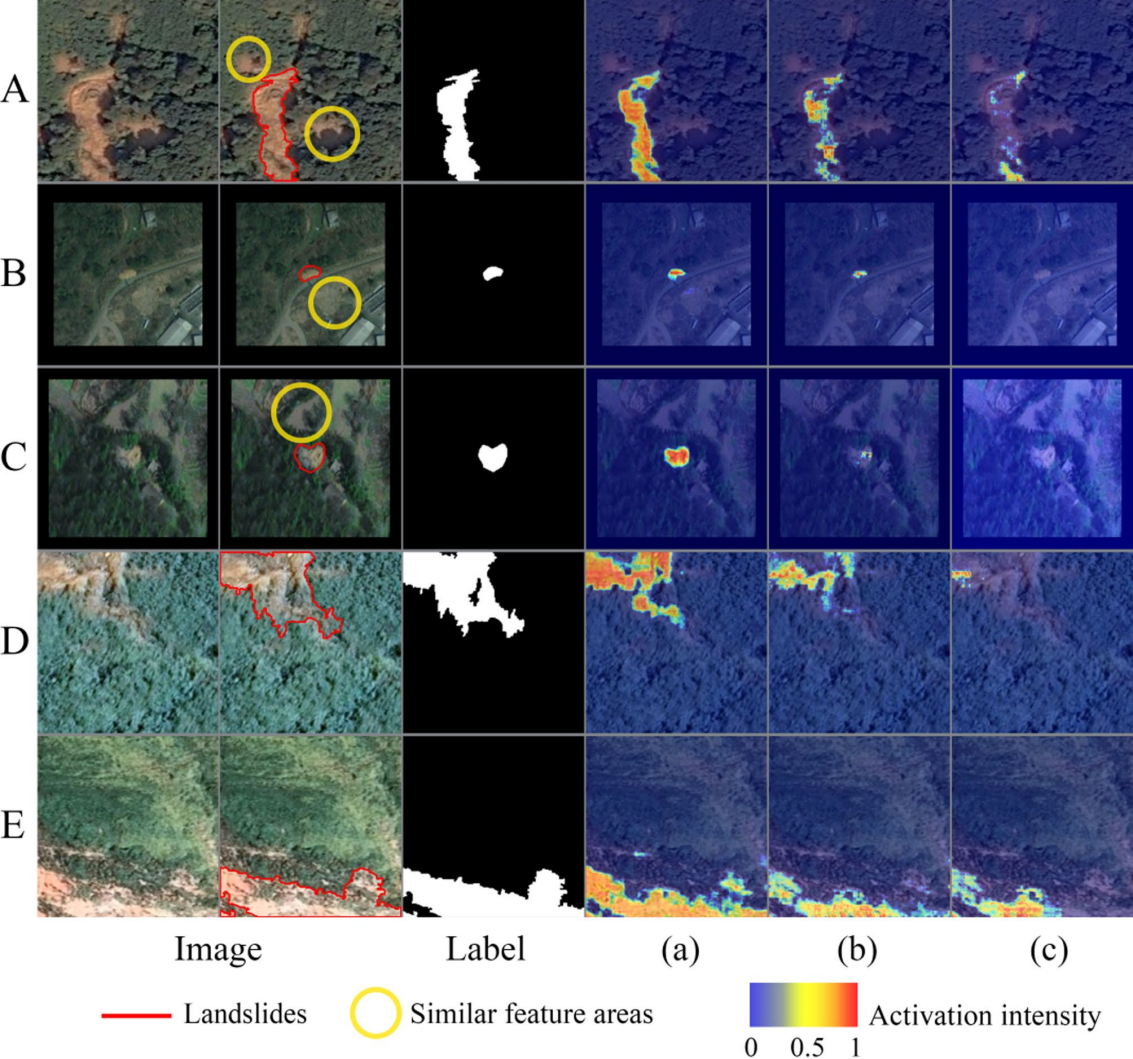
Grad-CAM visualization results for the validation set samples in the ablation study. (**a**) Baseline + MLP Head + CAB; (**b**) Baseline + MLP Head; (**c**) Baseline. (The visualization was implemented using Python 3.8.18 (https://www.python.org/), and the figure panels were laid out using Adobe Photoshop 22.5.4 (https://www.adobe.com/)).

The Baseline + MLP Head + CAB combination not only improved the Baseline model’s shortcomings—such as missed detection in small landslide regions (sample B) and severe boundary omissions (sample D)—but also enhanced boundary continuity and spatial consistency. This optimization led to superior geometric integrity performance in complex scenarios (samples A and E), comprehensively surpassing the fragmented contour segmentation effects of the Baseline model.

As Fig. 6 showing, the analysis based on Grad-CAM generated decision region maps reveals that the Baseline + MLP Head + CAB model, which incorporates the cross-attention mechanism, demonstrates significant performance in decision region accuracy. Specifically, the red high response regions exhibit a more precise spatial correspondence with the sample labels.

The introduction of the CAB enhances the spatial alignment between the model predicted landslide decision regions and their true boundaries. For landslide samples B and C, which share similar morphological features, the Baseline + MLP Head + CAB architecture generates spatially concentrated attention weights, precisely focusing on the landslide areas. Even for the more geometrically challenging samples D and E, the model maintains spatially coherent and uniformly distributed attention.

In contrast, the Baseline model performs poorly: its attention patterns exhibit noticeable fragmentation and dispersion. Not only does it completely overlook the critical morphological features of samples B and C, but it also captures only a few scattered fragmentary features in sample D.

### Comparative experiments

#### Quantitative comparison

Table 5 provides a detailed listing of the evaluation results for various models on the validation set using the landslide segmentation metric. In this comparative experiment, the CALandDet model performed exceptionally well, achieving the highest *IoU* (82.65%) and *F*1 score (81.64%). Additionally, the CALandDet model also recorded commendable results in terms of *P* (82.95%) and *R* (80.38%), securing the second highest rankings in both metrics.

**Table 5:** The evaluation of the validation set in the comparative experiments.

Model name	*IoU *(%)	*F*1 (%)	*P* (%)	*R* (%)
U-Net^43^	72.50	73.02	**86.92**	62.95
LinkNet^44^	71.95	80.97	79.38	**82.63**
DeepLab v3 + ^45^	71.87	79.03	82.32	75.99
SegFormer^8^	74.60	77.09	80.49	73.97
CALandDet	**82.65**	**81.64**	82.95	80.38

#### Visual comparison

**Fig. 7:**
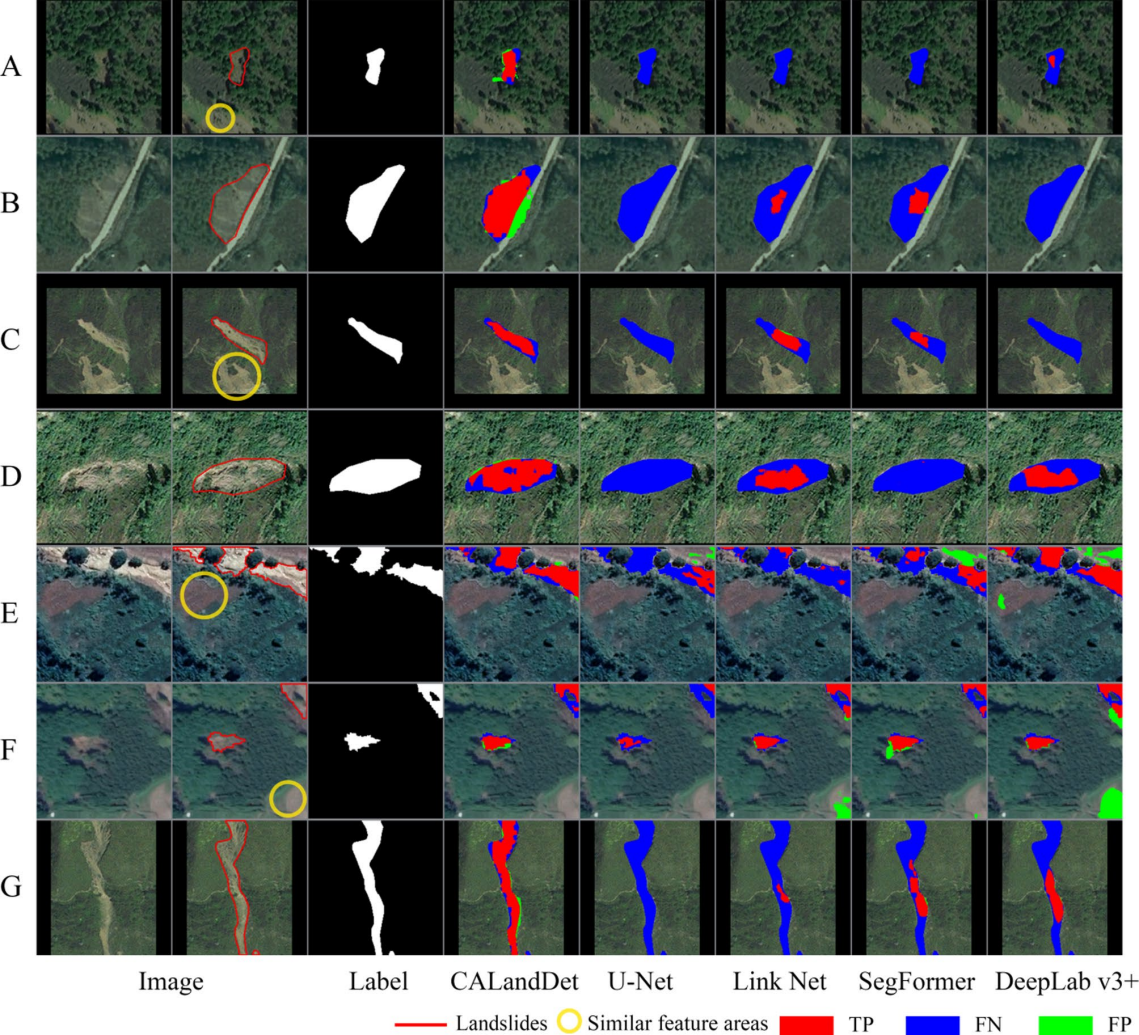
Visualization of recognition results for the validation set in comparative experiments (the visualization was implemented using Python 3.8.18 (https://www.python.org/), and the figure panels were laid out using Adobe Photoshop 22.5.4 (https://www.adobe.com/)).

Figure 7 displays the landslide segmentation performance of five models on the validation set. For this study, seven representative samples were selected for a detailed comparison, and the results were overlaid on remote sensing images. In samples A to D, due to the landslide features being texturally similar to and blurred with the background, the other four models exhibited varying degrees of missed detections. However, the CALandDet model was able to effectively recognize landslide features and accurately delineate the landslide boundaries, which corresponds with its higher *R* rate. For landslide samples E and F, which are located at the edges, the CALandDet model was still able to accurately recognize and thoroughly describe the landslide locations. In contrast, the other four models showed poorer recognition performance at these edge locations. Furthermore, for the elongated landslide sample G, the CALandDet model demonstrated superior recognition performance, surpassing the other models.

Although segmentation performance varies among different models, they universally face two critical challenges: (1) When landslide areas are intermixed with surrounding environments (dense vegetation with similar spectral or textural characteristics), models struggle to accurately delineate landslide boundaries. This typically manifests as boundary diffusion (blurred contours) and the generation of internal voids. For instance, in sample D, the CALandDet model exhibited such errors. (2) Systematic segmentation degradation occurs in edge regions of images when landslides are located near image boundaries due to the lack of contextual information. As demonstrated in samples E and F, this leads to blurred landslide contours, fragmented identification areas, or over-segmentation across all models—even under clear boundary conditions. Notably, models like U-Net and LinkNet showed particularly pronounced performance degradation in sample E.

**Fig. 8:**
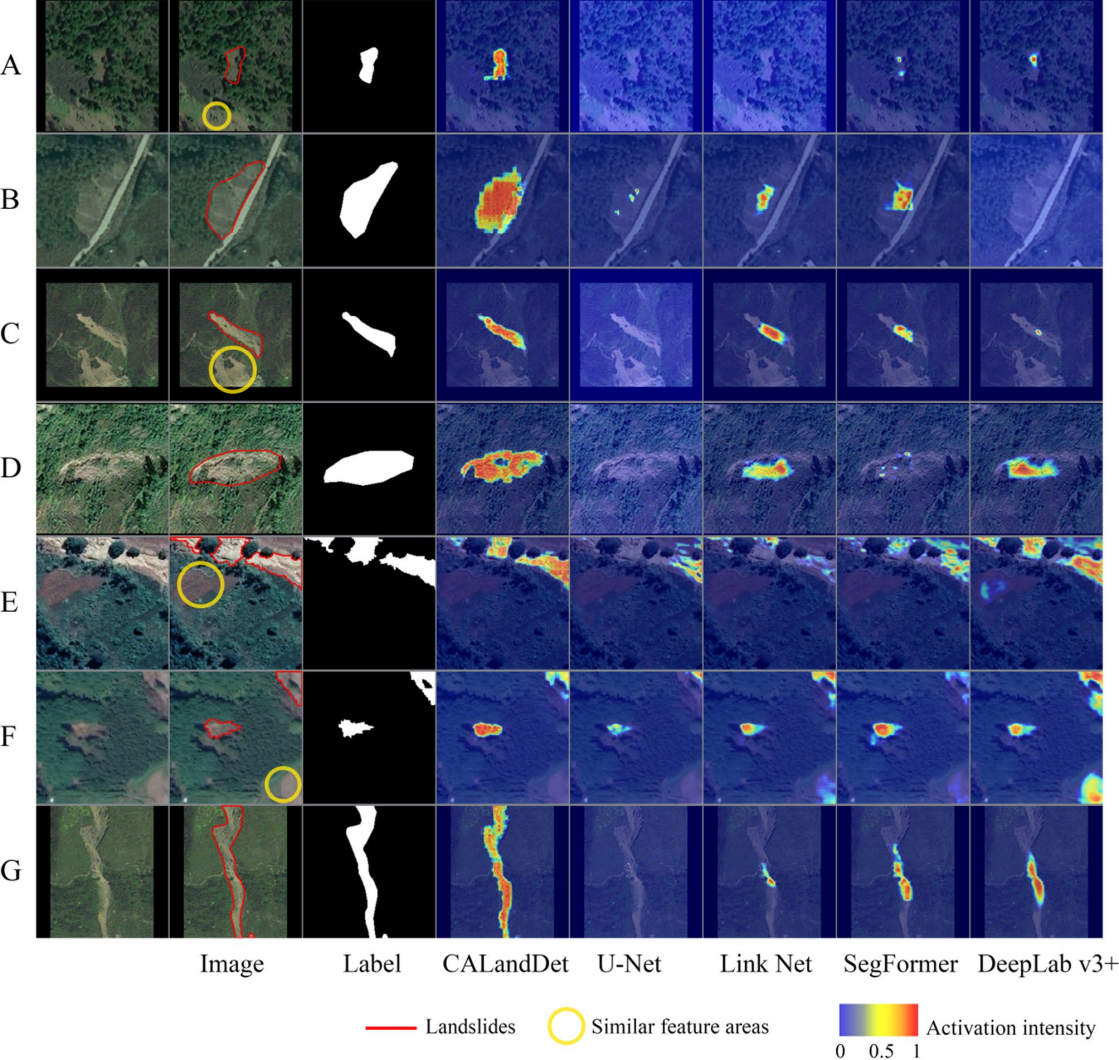
Visualization of Grad-CAM results for sample validation set in comparative experiments (the visualization was implemented using Python 3.8.18 (https://www.python.org/), and the figure panels were laid out using Adobe Photoshop 22.5.4 (https://www.adobe.com/)).

As Fig. 8 shows, the experimental results demonstrate that CALandDet significantly outperforms the comparative models in terms of landslide region identification accuracy. Through visual analysis, it is evident that the decision regions generated by CALandDet exhibit a higher degree of spatial consistency with the true landslide regions. The red high response areas show a more precise correspondence with the sample labels. Specifically, for challenging small sample cases such as Sample A, where the lower part of the sample contains similar feature information, CALandDet not only accurately captures the critical landslide features but also effectively avoids misidentification. For Sample B, which exhibits weaker landslide characteristics, the model’s decision regions still maintain a high response to the true landslide areas. Additionally, for Sample G, which contains a uniquely shaped elongated landslide, CALandDet demonstrates exceptional recognition capabilities.

## Discussion

As shown in Table 5, the proposed CALandDet model demonstrates significant advantages in landslide detection performance. Compared to baseline models, it achieves an 8.05–10.78% improvement in *IoU* and a 1.05–8.90% increase in *F*1 score. This performance enhancement primarily stems from the dual functionality of the CAB module: it effectively enhances the model’s capability to capture critical landslide identification features (such as surface texture, sliding surface debris, accumulation morphology, and vegetation destruction patterns) while simultaneously suppressing complex background interference. Ablation study results (Table 4) further confirm the pivotal role of CAB, where its removal leads to an 8.21% decrease in *IoU* and a 2.68% reduction in *F*1 score. These findings clearly indicate that CAB’s feature decoupling capability in complex geographical environments is crucial for the model’s overall performance, enabling CALandDet to demonstrate potential for high accuracy landslide identification in real-world scenarios. Additionally, we observed that removing the MLP Head also negatively impacts model accuracy, resulting in a 0.52% *IoU* decline and a 2.21% *F*1 score drop. This phenomenon may be attributed to the MLP Head’s direct connection to the loss function, which potentially accelerates gradient propagation and facilitates parameter optimization in the backbone network, providing valuable insights for future model architecture design.

Notably, although CALandDet leads in *IoU* and *F*1 score metrics, its *P* and *R* performance ranks second-best. This reveals the inherent Precision-Recall trade-off in landslide detection tasks—pursuing high *P* may lead to missed detections (low *R*), while emphasizing high *R* tends to introduce false positives (low *P*). Future work should explore adaptive confidence threshold mechanisms (e.g., region-specific threshold adjustment based on confidence distribution) and terrain confusion suppression strategies (e.g., enhancing spatial representation of key landslide features) to achieve synergistic improvement. Visualization results (Fig. 7) intuitively demonstrate the model’s strengths: CALandDet not only accurately delineates landslide boundaries (including slender shapes or small targets) but also exhibits strong discriminative power against spectrally-texturally similar terrain. However, current limitations remain, particularly in target separation under dense interference and performance degradation in regions lacking sufficient contextual information. Future improvements may incorporate multi-scale context aggregation mechanisms and semantically enhanced boundary optimization strategies to address these challenges.

Attention distribution analysis (Fig. 8) reveals that CALandDet’s decision regions exhibit higher spatial consistency with actual landslides. However, a missing response was observed in the upper-right landslide of sample F. In-depth analysis suggests that CAB’s reliance on global feature vectors generated by MLP Head for background suppression may cause dominant features to overshadow secondary targets in multi-landslide scenarios. For instance, when two landslide regions with distinct feature information (one exhibiting prominent features while the other displays relatively subtle characteristics) coexist within a sample, the dominant features of the conspicuous landslide body may overshadow the more subdued target, leading to ineffective detection of the latter. Additionally, it is imperative to account for the spatial autocorrelation articulated by Tobler’s First Law of Geography^46^. When the spatial scale of an individual sample (e.g., constrained by image resolution or sample size) exceeds the inherent spatial autocorrelation range of landslide features, the feature extraction paradigm adopted in this study may struggle to adequately resolve such spatial discontinuities or intra-class variations. Under such circumstances, the model not only fails to reconcile potential conflicts between target regions but may also suffer compromised overall recognition accuracy as a result. Future architectural optimizations could incorporate structured feature interaction frameworks (e.g., graph neural networks) to establish spatially aware feature fusion mechanisms, enhancing model adaptability across complex multi-scale scenarios.

While this study enhances the model’s generalization capability through multi-source dataset integration—spanning diverse geographical regions, geological contexts, and triggering mechanisms to capture universal landslide development patterns beyond localized features—critical challenges in data integration require systematic resolution. Inconsistent landslide interpretation criteria introduce label ambiguity, necessitating rigorous standardization to address feature scale and distribution heterogeneity across sources, alongside dedicated mitigation of spatial resampling artifacts and class imbalance. Furthermore, current datasets inadequately represent complex real-world scenarios involving cloud occlusion, extreme illumination variations, and multi-sensor discrepancies. These limitations, compounded by inherent domain shift in single-modal data, constrain practical deployment efficacy. Future work will develop a robust multi-modal framework featuring: (1) spatiotemporal registration and cross-scale feature matching to fuse optical imagery, time-series InSAR deformation metrics, SAR intensity/coherence data, and terrain-derived parameters, exploiting inter-modal complementarity; and (2) an adaptive input architecture enabling stable inference with available data combinations (e.g., partial optical/UAV imagery, extractable SAR measurements, and core topographic data) during critical scenarios such as large-area optical data gaps or InSAR processing failures.

## Conclusions

This study introduces a landslide segmentation approach that employs cross-attention feature enhancement mechanism to rapidly and accurately map landslides. Through comparative experiments with a baseline dataset, the following conclusions were drawn:

1. The proposed model demonstrates good landslide extraction accuracy and recognition performance. Compared to models such as U-Net, DeepLabv3+, LinkNet, and SegFormer, CALandDet’s *IoU* improved by 8.05–10.78%, and the *F*1 score increased by 1.05–8.9%. This is because CALandDet captures global landslide features via an MLP Head and enhances landslide feature discrimination while suppressing background noise through the use of a cross-attention feature enhancement mechanism. Through the combination of localized geomorphological signatures and global contextual features, this approach enables CALandDet to accomplish multi-scale feature fusion, which successfully identifies small or narrow landslides and enables more precise landslide boundary delineation.
2. The ablation experiments confirm that the cross-attention feature enhancement algorithm significantly enhances the effectiveness of landslide feature extraction: the *IoU* and *F*1 score of the Baseline + MLP Head + CAB model increased by 8.73% and 4.89%, respectively. By augmenting the representation capability of critical features and suppressing background noise interference, this algorithm effectively improves model performance, providing robust support for its application in landslide identification tasks.
3. Grad-CAM visualization analysis reveals that the decision regions generated by the CALandDet model exhibit higher spatial consistency with the true landslide regions, indicating that the model competently captures key features such as surface texture, sliding surface debris, accumulation bodies, and vegetation destruction. This provides theoretical support for the model’s performance.

Our proposed CALandDet landslide detection model demonstrates significant performance gains, particularly in *IoU* and *F*1 score metrics, primarily attributable to the dual functionality of the CAB module. This module effectively enhances discriminative landslide feature extraction while suppressing complex background interference, a critical mechanism validated through ablation studies. Despite these advancements, notable challenges persist. These include the inherent Precision-Recall trade-off requiring adaptive optimization strategies; instances of dominant landslides obscuring subtle targets in multi-target scenarios; and the model’s robustness under complex operational conditions, such as significant cloud occlusion or sensor disparities, which necessitates further rigorous validation. Future research will prioritize: (1) the development of spatially aware feature interaction frameworks (e.g., graph neural networks) to address multi-target conflicts and scale mismatches and (2) the construction of robust multi-modal collaborative learning systems adaptable to partial or heterogeneous input data (e.g., optical imagery, SAR, topographic data). These efforts aim to enhance the model’s practical applicability in challenging post-disaster environments, particularly when faced with incomplete or suboptimal data availability.

## Data Availability

The code used in the paper and the experimental dataset can be obtained from https://github.com/venjiehu/CALandDet.
